# Influence of Immediate Dentin Sealing on the Color Stability of Lithium Disilicate Ceramic Laminate Veneers Bonded to Sound and Eroded Dentin After Accelerated Aging

**DOI:** 10.3390/ma19163482

**Published:** 2026-08-18

**Authors:** Otavio Marino dos Santos Neto, Ingrid Carneiro Cavalcante Souto, Patrícia Pauletto, Rossana Pereira Almeida

**Affiliations:** 1 Department of Dental Materials and Prothesis, Ribeirão Preto School of Dentistry, University of São Paulo (USP), Ribeirão Preto 14040-902, SP, Brazil; otavio_marino@alumni.usp.br (O.M.d.S.N.); ingrid_ccsouto@hotmail.com (I.C.C.S.); rpaa@forp.usp.br (R.P.A.); 2 Department of Dentistry, Faculty of Dentistry, Universidad de las Americas (UDLA), Quito 170124, Ecuador

**Keywords:** immediate dentin sealing, lithium disilicate ceramics, laminate veneers, color stability, dentin erosion

## Abstract

**Highlights:**

- Immediate dentin sealing increased color change in lithium disilicate laminate veneers.
- Dentin erosion did not significantly affect Δ*E*_00_ values after accelerated aging.
- IDS-related color change was mainly associated with reduced luminosity.
- All groups exceeded perceptibility and acceptability thresholds after thermocycling.

**Abstract:**

The optical stability of thin ceramic laminate veneers may be affected by both the adhesive interface and the underlying substrate. This in vitro study evaluated the influence of immediate dentin sealing (IDS) on the color stability of lithium disilicate laminate veneers bonded to sound and eroded dentin after accelerated aging. Forty bovine dentin specimens were allocated into four groups according to dentin condition and adhesive protocol: sound dentin without IDS, sound dentin with IDS, eroded dentin without IDS, and eroded dentin with IDS. Lithium disilicate disks were cemented using a light-cured resin cement. Color coordinates were measured with a spectrophotometer before and after thermocycling, and color change was calculated using the CIEDE2000 formula (Δ*E*_00_). Changes in ΔL*, Δa*, and Δb* were also analyzed. Data were analyzed using two-way ANOVA and Tukey’s test (α = 0.05). IDS significantly increased Δ*E*_00_ values (4.63 ± 0.89) compared with the NoIDS groups (2.51 ± 0.49) (*p* < 0.001), whereas dentin condition and the interaction were not significant. IDS also affected ΔL*, Δa*, and Δb*, with the greatest change observed for ΔL*. All groups exceeded perceptibility and acceptability thresholds. IDS increased color change in lithium disilicate laminate veneers after accelerated aging, regardless of dentin condition.

## 1. Introduction

Ceramic laminate veneers are widely used for minimally invasive esthetic rehabilitation because they combine favorable optical properties, biocompatibility, and long-term clinical performance [1,2,3]. Advances in adhesive systems, resin cements, and glass-ceramic materials have enabled the fabrication of thinner restorations while preserving dental structure and providing predictable esthetic outcomes. However, because of their reduced thickness and high translucency, the final appearance of ceramic laminate veneers depends not only on the ceramic material itself, but also on the optical characteristics of the underlying dental substrate and the materials used during adhesive cementation [4,5,6,7].

Previous studies have shown that restoration thickness, ceramic translucency, substrate shade, and resin-based luting materials can influence the final color and color stability of laminate veneers [4,5,6,7,8]. Resin cements may produce perceptible color alterations, particularly when used under thin and highly translucent ceramic restorations [4,5]. In addition, aging of resin-based materials may compromise long-term optical stability through changes in luminosity and chromatic coordinates [5,6,7]. Although most investigations have focused on resin cements, adhesive systems may also affect the optical behavior of thin ceramic restorations, especially after aging [8,9]. This effect may be related to adhesive composition, photoinitiators, hydrophilic monomers, water sorption, and hydrolytic degradation of the bonded interface [9,10,11,12].

The increasing prevalence of erosive tooth wear has expanded the need for conservative restorative strategies capable of restoring esthetics and function while preserving the remaining dental tissues [13,14]. Ceramic laminate veneers may be indicated for teeth affected by erosive wear because they allow esthetic rehabilitation with limited additional tooth reduction [15]. Nevertheless, erosive processes can modify dentin morphology and composition, increasing surface porosity, collagen exposure, and permeability, which may affect adhesive procedures and the long-term behavior of bonded restorations [13,14,16].

Immediate dentin sealing (IDS) has been recommended for indirect adhesive restorations because it may improve bond strength, reduce dentin permeability, protect freshly prepared dentin, and enhance the quality of the adhesive interface [17,18,19,20,21]. The technique consists of applying and polymerizing an adhesive system immediately after tooth preparation, before provisionalization and definitive cementation. Despite these adhesive advantages, IDS creates an additional resin-based layer between the dentin substrate and the ceramic restoration. In thin laminate veneers, adhesive systems and resin-based interfaces may influence light transmission, scattering, and absorption through the restorative complex [8,9]. In addition, hydrophilic components, water sorption, and hydrolytic degradation of the bonded interface may contribute to optical changes after aging [10,11,12]. Therefore, although IDS is generally considered beneficial from a bonding perspective, its optical effect remains a relevant concern when highly translucent ceramic restorations are bonded to exposed dentin.

Evidence regarding the effect of IDS on the color stability of lithium disilicate laminate veneers bonded to sound and eroded dentin remains limited. Thus, the aim of this in vitro study was to evaluate the influence of IDS on the color stability of lithium disilicate ceramic laminate veneers bonded to sound and eroded dentin after accelerated aging. The research hypotheses were that IDS would affect color stability, dentin erosion would affect color stability, and the effect of IDS would depend on dentin substrate condition. The principal finding was that IDS increased color change after accelerated aging, regardless of dentin condition, whereas dentin erosion did not significantly affect Δ*E*_00_ values.

## 2. Materials and Methods

### 2.1. Study Design and Sample Size Calculation

This in vitro study evaluated the influence of immediate dentin sealing (IDS) and dentin substrate condition on the color stability of lithium disilicate ceramic laminate veneers after accelerated aging. The experimental factors were dentin condition, at two levels—sound dentin and eroded dentin—and adhesive protocol, at two levels—with or without IDS. The response variables were color difference calculated using the CIEDE2000 formula (Δ*E*_00_) and changes in the CIELAB coordinates (ΔL*, Δa*, and Δb*).

Sample size calculation was performed using Power and Sample Size Calculation software (version 3.1.6; Vanderbilt University, Nashville, TN, USA), based on preliminary data obtained from a pilot study. Color change, expressed as Δ*E*_00_, was defined as the primary outcome. The pilot study indicated an expected difference of 1.40 Δ*E*_00_ units between the adhesive protocols, with a pooled standard deviation of 1.05, corresponding to a standardized effect size of 1.33. Considering a statistical power of 80%, a two-sided significance level of 5%, and equal group sizes, the calculation indicated that 10 specimens per experimental group were required to detect the expected difference between the adhesive protocols.

**Figure 1:**
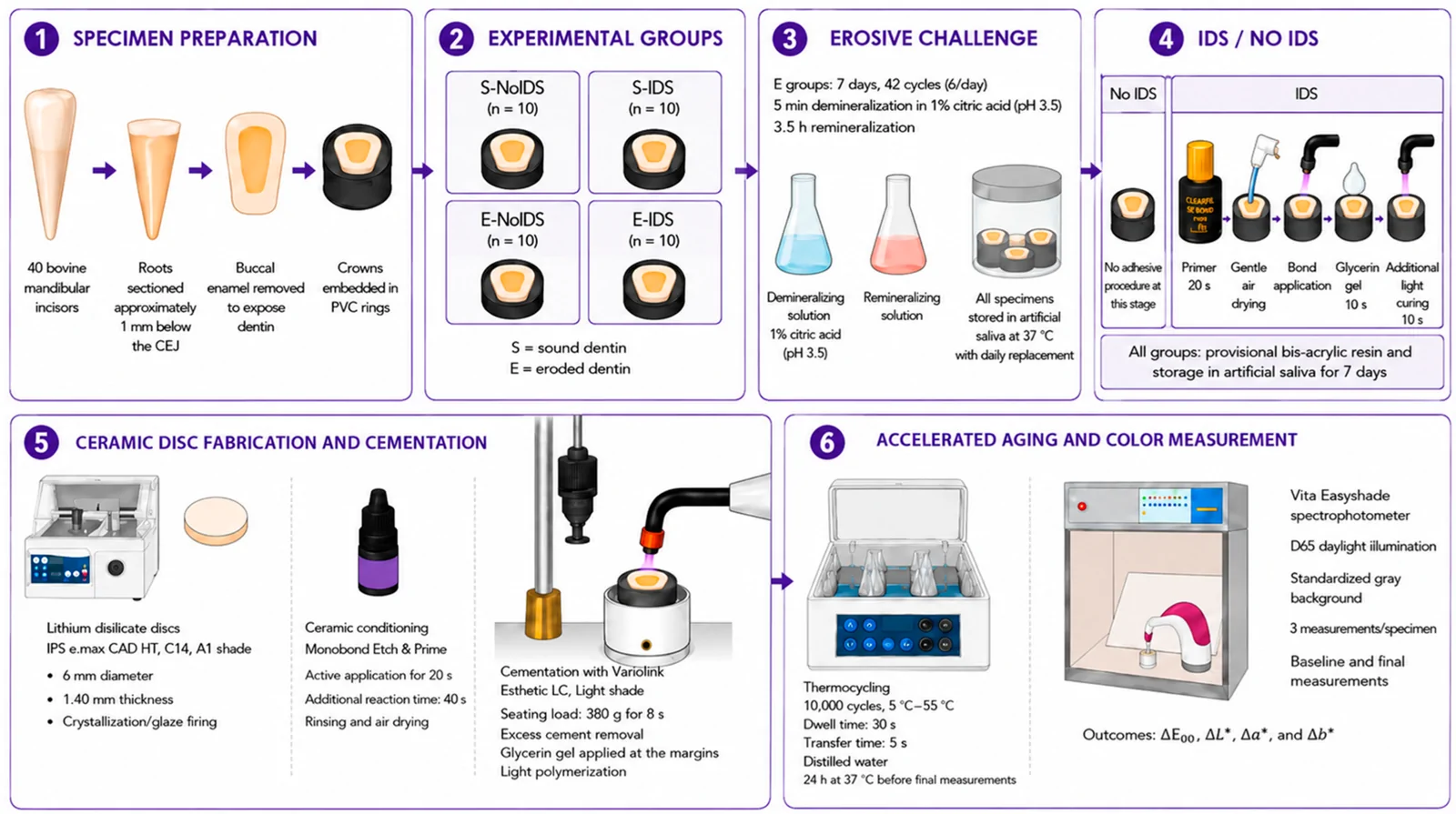
Experimental workflow.

Therefore, 40 specimens were allocated into four experimental groups (n = 10): S-NoIDS, sound dentin without IDS; S-IDS, sound dentin with IDS; E-NoIDS, eroded dentin without IDS; and E-IDS, eroded dentin with IDS. The experimental workflow is presented in Figure 1, which was prepared with the assistance of ChatGPT (OpenAI, GPT-5.5 Thinking) based on text prompts provided by the authors describing the experimental procedures. The AI-assisted schematic was subsequently reviewed, corrected, edited, and finalized by the authors to ensure scientific accuracy and consistency with the methodology. No copyrighted third-party images or previously published figures were used as input.

### 2.2. Ethical Considerations

This in vitro study used bovine teeth obtained from animals slaughtered for reasons unrelated to this research. No live animals or human participants were involved. Therefore, formal ethical approval was not required according to institutional regulations. The teeth were collected after slaughter and handled in accordance with biosafety procedures.

### 2.3. Materials

The materials used in this study, including commercial names, manufacturers, compositions, and lot numbers, are listed in Table 1.

### 2.4. Tooth Selection and Specimen Preparation

Freshly extracted bovine mandibular incisors free of cracks, structural defects, or visible surface irregularities were selected. Organic debris was removed with periodontal curettes, and the teeth were stored in 1% chloramine-T solution until specimen preparation.

The roots were sectioned approximately 1 mm below the cementoenamel junction using a precision cutting machine under water cooling. The crowns were then stored in deionized water at 4 °C until preparation. To simulate laminate veneer preparations involving dentin exposure, the buccal enamel surface was removed using 120-grit silicon carbide abrasive paper in a metallographic polishing machine until dentin was exposed. The crowns were subsequently reduced to standardized dimensions defined by polyvinyl chloride rings and embedded in autopolymerizing acrylic resin, leaving the prepared buccal dentin surface exposed.

The specimens were stored in artificial saliva at 37 °C for 7 days before the experimental procedures, with daily replacement of the solution. Artificial saliva consisted of 0.33 g KH_2_PO_4_, 0.34 g Na_2_HPO_4_, 1.27 g KCl, 0.16 g NaSCN, 0.58 g NaCl, 0.17 g CaCl_2_, 0.16 g NH_4_Cl, 0.20 g urea, 0.03 g glucose, and 0.002 g ascorbic acid per liter, at pH 7.0.

### 2.5. Ceramic Specimen Preparation

Seven lithium disilicate CAD/CAM blocks (IPS e.max CAD HT, C14, A1 shade; Ivoclar Vivadent, Schaan, Liechtenstein) were used. To obtain standardized cylindrical specimens, the blocks were machined in a universal grinding machine to produce cylinders measuring 6 mm in diameter. The cylinders were then sectioned under water cooling using a diamond wafering blade mounted on a precision cutting machine (Isomet 1000, Buehler, IL, USA), producing ceramic disks with an approximate thickness of 1.40 mm.

Before crystallization, disk thickness was measured using a digital caliper, and specimens presenting cracks, defects, or visible imperfections were diskarded. A glaze layer (IPS Ivocolor Glaze Fluo Paste, Ivoclar Vivadent, Schaan, Liechtenstein), diluted with IPS Ivocolor Mixing Liquid Allround (Ivoclar Vivadent, Schaan, Liechtenstein), was applied to one surface of each disk. The specimens underwent a single crystallization/glaze firing cycle in a ceramic furnace (Programat EP5010, Ivoclar Vivadent, Schaan, Liechtenstein) according to the manufacturer’s recommendations. After firing, the thickness of each disk was reassessed using a digital caliper to account for dimensional changes associated with ceramic densification. Specimens that did not present a final thickness of 1.40 mm or that exhibited cracks, defects, or visible imperfections were discarded. A total of 40 ceramic disks meeting the inclusion criteria were used in the study.

### 2.6. Erosive Challenge

Specimens assigned to the E-NoIDS and E-IDS groups were subjected to an erosive cycling protocol adapted from a previously described method [22]. The compositions of the demineralizing and remineralizing solutions are presented in Table 2.

A total of 42 demineralization/remineralization cycles were performed over 7 days, with 6 cycles per day. Each cycle consisted of 5 min of immersion in the demineralizing solution followed by 3.5 h of immersion in the remineralizing solution. Between cycles, specimens were rinsed with deionized water. The pH of both solutions was monitored daily, and the solutions were replaced every 24 h.

After the erosive challenge, the specimens were stored in artificial saliva at 37 °C with daily replacement of the solution until the adhesive procedures were performed. Specimens assigned to the S-NoIDS and S-IDS groups were maintained in artificial saliva under the same conditions throughout the experimental period.

### 2.7. Immediate Dentin Sealing and Provisionalization

Before the adhesive procedures, all dentin surfaces were polished with 600-grit silicon carbide abrasive paper for 20 s under water cooling to produce a standardized smear layer. This polishing protocol was adopted to standardize the smear layer before the adhesive procedures. A pilot evaluation using confocal laser scanning microscopy demonstrated that polishing for 20 s did not eliminate the surface characteristics produced by the erosive challenge, thereby preserving the eroded dentin morphology.

For the S-IDS and E-IDS groups, dentin surfaces were cleaned with a slurry of pumice and water applied with a Robinson brush, rinsed with water spray, and air-dried. The primer of a two-step self-etch adhesive system (Clearfil SE Bond, Kuraray Noritake, Osaka, Japan) was actively applied for 20 s, followed by gentle air drying. The bonding agent was then applied in a uniform layer and photoactivated for 10 s using an LED curing unit (Bluephase N, Ivoclar Vivadent, Schaan, Liechtenstein) at an irradiance of 1200 mW/cm^2^. To eliminate the oxygen-inhibited layer, a glycerin-based gel (Power Block, Maquira, Maringá, Brazil) was applied over the adhesive surface, followed by an additional 10 s photoactivation cycle. No adhesive procedure was performed for the S-NoIDS and E-NoIDS groups at this stage.

To simulate provisional restorations and standardize the interval between dentin preparation and definitive cementation, bis-acrylic resin (Primma Art A2, FGM, Joinville, Brazil) was applied over the dentin surfaces of all specimens. The specimens were stored in artificial saliva at 37 °C for 7 days, with daily replacement of the solution.

### 2.8. Cementation Procedure

The non-glazed ceramic surface was treated with Monobond Etch & Prime (Ivoclar Vivadent, Schaan, Liechtenstein) according to the manufacturer’s instructions. The material was actively applied to the ceramic surface for 20 s, followed by an additional reaction time of 40 s. The surface was then thoroughly rinsed with water until the characteristic green coloration was completely removed and dried with oil-free compressed air.

After removal of the provisional material, dentin surfaces in all groups, including the previously sealed IDS specimens, were cleaned with a slurry of pumice and water applied with a Robinson brush to remove provisional material remnants, rinsed with water spray, and air-dried. No additional mechanical surface treatment was performed before the final adhesive procedure. For both IDS and NoIDS groups, the primer of the adhesive system (Clearfil SE Bond, Kuraray Noritake, Osaka, Japan) was actively applied for 20 s, followed by gentle air drying. The bonding agent was applied in a thin layer without photoactivation.

A light-cured resin cement (Variolink Esthetic LC, Light shade; Ivoclar Vivadent, Schaan, Liechtenstein) was applied to the conditioned ceramic surface, and the ceramic disks were positioned centrally over the dentin substrate. A dental surveyor was used to standardize a seating load of 380 g for 8 s during cementation. Excess cement was removed with disposable microbrushes and exploratory probes. A glycerin gel (Power Block, Maquira, Maringá, Brazil) was applied around the restoration margins to prevent the formation of an oxygen-inhibited layer.

Polymerization was performed using an LED curing unit (Bluephase N, Ivoclar Vivadent, Schaan, Liechtenstein) at an irradiance of 1200 mW/cm^2^, according to the manufacturer’s recommendations. The specimens were stored in distilled water at 37 °C for 7 days before baseline color measurements.

### 2.9. Accelerated Aging

Baseline color measurements were obtained after completion of the cementation procedures. The specimens were subsequently subjected to 10,000 thermocycles between 5 °C and 55 °C, with a dwell time of 30 s in each bath and a transfer time of 5 s between baths. Distilled water was used as the bath medium. After thermocycling, the specimens were stored in distilled water at 37 °C for 24 h before the final color measurements were performed.

### 2.10. Color Evaluation

Color measurements were performed with a spectrophotometer (Vita Easyshade, Vita Zahnfabrik, Bad Säckingen, Germany) before and after thermocycling. Specimens were maintained moist during measurements, and reference marks were used to standardize positioning of the spectrophotometer tip. The tip was positioned at the center of each ceramic disk, perpendicular to the ceramic surface, and away from the specimen margins. The spectrophotometer was calibrated according to the manufacturer’s instructions before each measurement session.

Measurements were obtained in a metamerism box simulating daylight illumination (D65; GTI MiniMatcher, GTI Graphic Technology Inc., Newburgh, NY, USA). A custom-made gray mask, fabricated from the same material as the gray background, was adapted over each specimen during color measurements. The mask contained a central aperture that exposed only the cemented ceramic restoration while covering the surrounding acrylic resin and polyvinyl chloride ring. Thus, all readings were performed against a standardized gray background with CIELAB coordinates of L* = 72.4, a* = −0.05, and b* = 1.25. Three measurements were recorded for each specimen, and the mean L*, a*, and b* values were used for analysis.

Color difference was calculated using the CIEDE2000 formula (Δ*E*_00_) with dedicated software (i7 Delta Color, version 1.0.5.5., São Leopoldo, Brazil). Changes in the CIELAB coordinates were calculated as ΔL* = Lf − Li, Δa* = af − ai, and Δb* = bf − bi, where i and f represent the initial and final measurements, respectively. The 50:50% perceptibility threshold (PT = 0.8) and acceptability threshold (AT = 1.8) were adopted for the interpretation of color differences [23].

### 2.11. Statistical Analysis

Data distribution was assessed using the Shapiro–Wilk test, and homogeneity of variance was evaluated using Levene’s test. The effects of dentin condition and adhesive protocol, as well as their interaction, on Δ*E*_00_, ΔL*, Δa*, and Δb* were analyzed using two-way analysis of variance. When significant effects were detected, Tukey’s post hoc test was used for multiple comparisons. All analyses were performed at a significance level of 5% (α = 0.05).

## 3. Results

### 3.1. Color Change (ΔE_00_)

**Figure 2:**
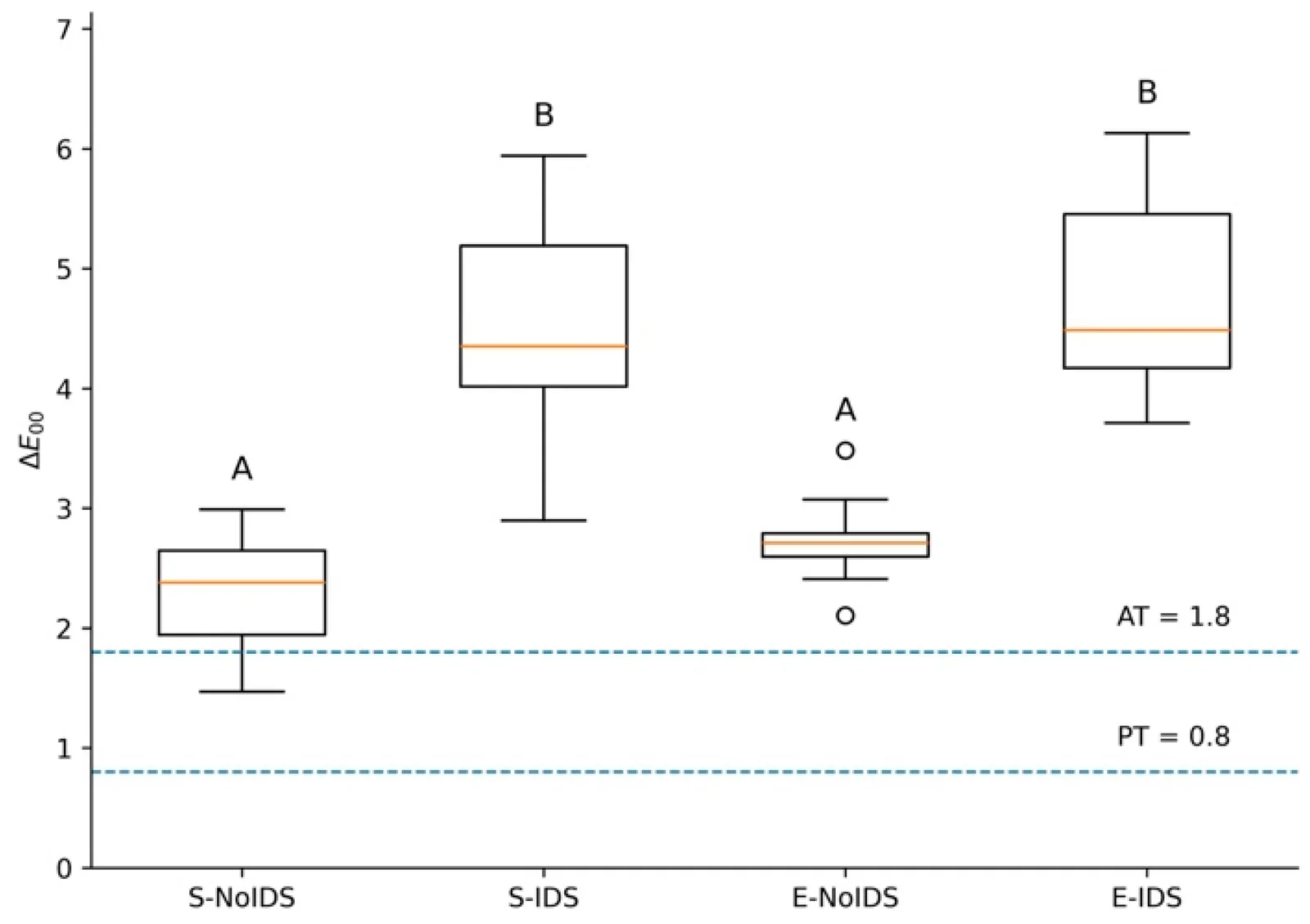
Distribution of Δ*E*_00_ values according to dentin condition and adhesive protocol. Boxplots represent the median, interquartile range, and minimum/maximum values, while circles indicate outliers. Horizontal dashed lines indicate the 50:50% perceptibility threshold (PT = 0.8) and acceptability threshold (AT = 1.8). Different uppercase letters indicate statistically significant differences between the levels of the IDS factor (Tukey test, α = 0.05).

Mean and standard deviation values of Δ*E*_00_ are presented in Table 3, whereas the complete two-way ANOVA results are shown in Table 4. The distribution of values among the experimental groups is presented in Figure 2.

Two-way ANOVA showed no significant main effect of dentin condition on Δ*E*_00_, F(1, 36) = 2.855, *p* = 0.100, ηp^2^ = 0.073, and no significant interaction between dentin condition and IDS, F(1, 36) = 0.075, *p* = 0.785, ηp^2^ = 0.002. In contrast, IDS significantly affected color change, F(1, 36) = 89.938, *p* < 0.001, ηp^2^ = 0.714.

Specimens restored using IDS showed significantly greater Δ*E*_00_ values than those restored without IDS, regardless of dentin condition. The mean Δ*E*_00_ value was 4.63 ± 0.89 for the IDS groups and 2.51 ± 0.49 for the NoIDS groups. The estimated mean difference between the adhesive protocols was 2.12 Δ*E*_00_ units (95% CI: 1.67 to 2.57). Numerically, the highest Δ*E*_00_ value was observed in the E-IDS group, whereas the lowest value was observed in the S-NoIDS group; however, the statistically significant difference was associated with the main effect of IDS.

Based on the adopted 50:50% perceptibility threshold (PT = 0.8) and acceptability threshold (AT = 1.8), all experimental groups presented Δ*E*_00_ values above both thresholds. Therefore, the color changes observed after accelerated aging exceeded the adopted perceptibility and acceptability thresholds, regardless of dentin condition or adhesive protocol.

### 3.2. Changes in CIELAB Coordinates

Baseline and final L*, a*, and b* values are presented in Table 5. Baseline measurements were obtained after cementation and before thermocycling, whereas final measurements were obtained after accelerated aging. Mean and standard deviation values of ΔL*, Δa*, and Δb* are presented in Table 6, and the complete two-way ANOVA results, including effect estimates and 95% confidence intervals, are shown in Table 7.

Across the experimental groups, accelerated aging was predominantly associated with a reduction in L* values, indicating decreased luminosity. Changes in a* and b* were smaller and varied according to dentin condition and adhesive protocol.

For ΔL*, dentin condition did not significantly affect the results, F(1, 36) = 2.628, *p* = 0.1138, ηp^2^ = 0.068, and no significant interaction was observed between dentin condition and IDS, F(1, 36) = 0.335, *p* = 0.5660, ηp^2^ = 0.009. However, IDS significantly influenced luminosity change, F(1, 36) = 53.428, *p* < 0.0001, ηp^2^ = 0.598. Specimens restored using IDS showed more negative ΔL* values than those restored without IDS, indicating a greater reduction in luminosity after accelerated aging.

For Δa*, significant main effects were observed for dentin condition, F(1, 36) = 9.927, *p* = 0.0033, ηp^2^ = 0.216, and IDS, F(1, 36) = 9.583, *p* = 0.0038, ηp^2^ = 0.210, whereas the interaction between the factors was not significant, F(1, 36) = 0.329, *p* = 0.5700, ηp^2^ = 0.009. Eroded dentin specimens showed higher Δa* values than sound dentin specimens, and the IDS groups showed higher Δa* values than the NoIDS groups.

For Δb*, IDS significantly affected the results, F(1, 36) = 6.712, *p* = 0.0137, ηp^2^ = 0.157, whereas dentin condition, F(1, 36) = 1.681, *p* = 0.2030, ηp^2^ = 0.045, and the interaction between the factors, F(1, 36) = 0.154, *p* = 0.6970, ηp^2^ = 0.004, were not significant. Specimens restored using IDS showed higher Δb* values than those restored without IDS.

Among the individual CIELAB coordinates, the greatest magnitude of change was observed for ΔL*. Thus, the greater color alteration observed in the IDS groups was mainly associated with reduced luminosity, indicating darkening of the restorative complex after accelerated aging.

## 4. Discussion

The first research hypothesis was supported because immediate dentin sealing (IDS) significantly affected the color stability of lithium disilicate ceramic laminate veneers after accelerated aging. Conversely, the second and third research hypotheses were not supported, as dentin erosion did not significantly influence Δ*E*_00_ values and the effect of IDS on color stability did not depend on dentin substrate condition. Evidence regarding the influence of IDS on the color stability of ceramic laminate veneers bonded to eroded dentin remains limited, which restricts direct comparisons with previous investigations. Under the experimental conditions of the present study, the adhesive protocol had a greater influence on color change than the dentin substrate condition.

The most relevant finding of the present investigation was that specimens restored using IDS exhibited significantly greater color change than those restored without IDS, regardless of dentin condition. This observation is particularly relevant because IDS has been extensively investigated with respect to bond strength, dentin permeability, postoperative sensitivity, and restoration longevity, with consistently favorable outcomes reported in laboratory and clinical studies [17,18,19,20,21]. However, the optical consequences of this technique have received considerably less attention. The present results suggest that, although IDS provides recognized adhesive benefits, the adhesive interface created by this protocol may also influence the long-term optical stability of thin ceramic restorations.

The influence of resin-based materials on the final color and color stability of ceramic laminate veneers has been previously reported [4,5,6,7,8,9,24,25]. Thin and highly translucent ceramic restorations are particularly susceptible to the optical influence of underlying materials, including the dental substrate, resin cement, and adhesive interface. Previous investigations have shown that adhesive systems may influence color matching and that different adhesive protocols may affect the color stability of thin ceramic veneers after aging [8,9,26]. In addition, resin-based luting materials and associated factors may substantially influence the color behavior of ceramic laminate veneers [4,5]. Collectively, these findings support the hypothesis that optical changes occurring within the adhesive interface may have contributed to the greater color alteration observed in the IDS groups.

One possible explanation for this behavior is the presence of an additional polymerized adhesive layer created by the IDS protocol between the ceramic restoration and the dentin substrate. Thus, the greater color change observed in the IDS groups may be associated with this additional resin-based layer rather than with dentin sealing itself. Previous studies have demonstrated that adhesive systems may undergo water sorption, solvent loss, hydrolytic degradation, and structural changes over time, potentially altering their optical properties and the stability of the bonded interface [10,11,12,27,28,29]. However, adhesive-layer thickness, water sorption, optical properties, and interfacial degradation were not directly measured in the present study. Therefore, these mechanisms should be regarded as possible explanations and not as direct evidence for the observed color changes. 

It is important to emphasize that the present findings reflect the specific IDS protocol evaluated in this study. Classic IDS protocols have often been associated with filled adhesive systems and, in some approaches, with an additional resin coating layer [17,18,19,20,21]. In the present investigation, IDS was performed using a two-step self-etch adhesive system without an additional flowable resin coating. This protocol was selected to simulate a conservative clinical situation involving thin laminate veneers and dentin exposure, in which increasing the thickness of the resin layer could interfere with the restorative space and internal adaptation. Moreover, adhesive performance may be influenced by adhesive composition, functional monomers, and etching strategy, which can affect the durability of the bonded interface [27,30,31,32,33,34]. Therefore, the present findings should not be generalized to all IDS strategies, adhesive systems, or resin-coating protocols.

The analysis of the individual CIELAB coordinates provides additional insight into the mechanisms responsible for the observed color changes. Although statistically significant differences were also detected for Δa* and Δb*, the greatest magnitude of change was observed for ΔL*. Specimens restored using IDS exhibited significantly greater reductions in luminosity than those restored without IDS. Therefore, the higher Δ*E*_00_ values observed in the IDS groups appear to be primarily associated with darkening of the restorative complex rather than pronounced alterations in hue or chroma. This finding suggests that the optical effect of IDS is predominantly related to changes in light transmission, scattering, or absorption through the ceramic-restoration complex after aging.

Dentin erosion did not significantly affect color stability. This finding contrasts with the extensive literature demonstrating that erosive tooth wear modifies dentin morphology and may compromise adhesive performance. Eroded dentin typically presents mineral loss, increased porosity, collagen exposure, and changes in substrate permeability, characteristics that may negatively affect bonding procedures [13,14,15,16,22,33,35,36]. Nevertheless, despite these structural alterations, no significant effect on Δ*E*_00_ was observed in the present study.

A possible explanation for the lack of a significant effect of dentin erosion on Δ*E*_00_ is that the optical contribution of the dentin substrate may have been reduced by the combined influence of the ceramic restoration, resin cement, and adhesive interface. Previous studies have shown that the final color of thin ceramic restorations is determined by the interaction among ceramic thickness, translucency, substrate color, resin cement, and the bonded substrate [4,5,37,38,39,40,41,42,43,44]. Furthermore, the absence of a statistically significant interaction between dentin condition and IDS suggests that the influence of IDS on color change was similar across both substrates under the experimental conditions evaluated. Thus, while dentin erosion remains highly relevant from a bonding perspective, its effect on the optical stability of ceramic laminate veneers appeared limited in the present experimental model. However, because the optical properties of the individual components and the bonded interface were not directly evaluated, and no microscopic, optical, or interfacial characterization was performed, these proposed mechanisms remain speculative and should be interpreted as possible explanations rather than direct evidence.

From a clinical perspective, the present findings should be interpreted cautiously. Numerous laboratory and clinical investigations have demonstrated that IDS may improve bond strength, reduce dentin permeability, decrease postoperative sensitivity, and enhance the clinical performance of indirect restorations [17,18,19,20,21,32,36,45]. Therefore, the greater color alteration observed in the present study should not be interpreted as evidence against the use of IDS. Rather, clinicians should recognize that the adhesive benefits associated with this technique may be accompanied by optical changes that become more evident in highly translucent ceramic restorations, particularly after aging. When thin restorations are bonded to exposed dentin, the optical behavior of the adhesive interface should be considered together with the biological and mechanical advantages of IDS. However, although IDS significantly increased Δ*E*_00_ values compared with the NoIDS groups, all experimental groups exceeded the adopted 50:50% acceptability threshold after accelerated aging. Therefore, the clinical relevance of this additional color change remains uncertain and should be confirmed in long-term clinical studies.

Another noteworthy finding was that all experimental groups exhibited Δ*E*_00_ values above both the perceptibility and acceptability thresholds proposed for dentistry [23]. This observation indicates that accelerated aging affected the optical behavior of the restorative complex regardless of dentin condition or adhesive protocol. Another noteworthy finding was that all experimental groups exhibited Δ*E*_00_ values above the adopted 50:50% perceptibility and acceptability thresholds. Similar findings have been reported in studies evaluating ceramic laminate veneers and other thin esthetic restorations, particularly when restoration thickness, ceramic translucency, substrate color, resin cement, and aging are considered [38,39,40,41,46,47,48,49,50,51,52]. However, because these thresholds were established under standardized experimental conditions, exceeding them should not be directly interpreted as evidence of clinical unacceptability. The Δ*E*_00_ values observed in the present study should therefore be interpreted within the broader optical complexity of thin ceramic restorations, in which subtle changes in the substrate and interposed resin-based materials may influence the final color. Nevertheless, the significantly greater color changes observed in the IDS groups suggest that the adhesive interface may intensify aging-related optical alterations. This interpretation is consistent with evidence that adhesive materials and aging-related degradation of the bonded interface can affect the optical behavior of restorative materials [8,9,10,11,12,27,28,29].

The present study has limitations. As an in vitro investigation, it does not fully reproduce the complexity of the oral environment, including biofilm accumulation, dietary pigments, salivary proteins, brushing abrasion, functional loading, and long-term intraoral aging. Furthermore, accelerated aging was limited to thermocycling, which does not encompass all mechanisms involved in the discoloration of resin-based materials [25,53]. In addition, only one ceramic material, one ceramic thickness, one resin cement, one adhesive system, and one IDS protocol were evaluated. Moreover, flat ceramic disks were used as a standardized experimental model and therefore do not fully reproduce the anatomical geometry and variable thickness of clinical laminate veneers. Bovine teeth were used as a substitute for human teeth because they provide a standardized and readily available substrate and have been widely employed in studies evaluating dental adhesion and the color stability of bonded ceramic restorations [54,55,56,57]. Nevertheless, structural and optical differences between bovine and human dentin should be considered when extrapolating the present findings to clinical situations. Additionally, the sample size calculation was not specifically powered to detect an interaction between IDS and dentin condition. Therefore, the non-significant interaction observed between these factors should be interpreted with caution, as the study may have had limited statistical power to detect an interaction effect. Accordingly, the results should not be generalized to all restorative systems or clinical conditions. Future studies should evaluate different adhesive systems, IDS protocols, ceramic thicknesses and translucencies, resin cement shades, and aging protocols. Additional investigations combining color analysis with optical, chemical, or interfacial characterization, as well as clinical studies, are needed to confirm the present findings under long-term oral conditions.

## 5. Conclusions

Within the limitations of this in vitro study, the following conclusions were drawn:

(1) The first research hypothesis was supported, as immediate dentin sealing significantly increased the color change of lithium disilicate ceramic laminate veneers after artificial aging, regardless of dentin condition.
(2) The second research hypothesis was not supported, as dentin erosion did not significantly affect Δ*E*_00_ values.
(3) The third research hypothesis was not supported, as no significant interaction was observed between dentin condition and IDS.
(4) The greater color alteration observed in the IDS groups was mainly associated with a reduction in luminosity, indicating darkening of the restorative complex after artificial aging.
(5) All experimental groups showed Δ*E*_00_ values above the adopted 50:50% perceptibility and acceptability thresholds after artificial aging.

## Figures and Tables

**Table 1:** Materials used in the study, manufacturers, compositions, and lot numbers.

Commercial Name/Product	Manufacturer	Composition	Lot Number
IPS e.max CAD Cerec/InLab HT, C14, A1 shade lithium disilicate CAD/CAM ceramic block	Ivoclar Vivadent	SiO_2_, Li_2_O, K_2_O, MgO, Al_2_O_3_, P_2_O_5_, and other oxides	Z00ZGP
Monobond Etch & Prime one-step ceramic primer	Ivoclar Vivadent	Aqueous solution of ammonium polyfluoride, silane methacrylate, and colorant	Z01C51
Clearfil SE Bond two-step self-etch adhesive system	Kuraray Noritake	Primer: 10-MDP, HEMA, hydrophilic dimethacrylate, dl-camphorquinone, N,N-diethanol-p-toluidine, and water. Bond: 10-MDP, Bis-GMA, HEMA, hydrophobic dimethacrylate, dl-camphorquinone, N,N-diethanol-p-toluidine, and silanated colloidal silica	00138
Variolink Esthetic LC light-cured resin cement, Light shade	Ivoclar Vivadent	Urethane methacrylate and methacrylate monomers; Ivocerin; ytterbium trifluoride and zirconium oxide spheres, 0.04–0.2 µm, mean size 0.1 µm, 38 vol%	Z014KC
Primma Art bis-acrylic resin, A2 shade	FGM	Base paste: UDMA and TEGDMA methacrylate monomers, coinitiators, barium-boroaluminosilicate glass microparticles, silicon dioxide particles, inorganic pigments, and stabilizers. Catalyst paste: methacrylate monomers, dibenzoyl peroxide, barium-boroaluminosilicate glass microparticles, and stabilizers	250220
Power Block water-soluble oxygen-blocking gel	Maquira	Carbopol, methylparaben, propylparaben, glycerin, propylene glycol, açaí flavoring, neutralizing agent, and purified water	466320
IPS Ivocolor Glaze Fluo Paste	Ivoclar Vivadent	Alkaline aluminosilicate glass and solvent	Z00VR0
IPS Ivocolor Mixing Liquid Allround	Ivoclar Vivadent	Solvents	Z021TF

**Table 2:** Composition of substances used for demineralization and remineralization.

Solution (37 °C)	Composition
Demineralization	Citric acid 1% with pH 3.5
Remineralization	0.002 g of ascorbic acid, 0.58 g of NaCl, 0.17 g of CaCl_2_, 0.16 g of NH_4_Cl, 1.27 g of KCL, 0.16 g of NaSCN, KH_2_PO_4_ 0, 34 g of Na_2_HPO_4_, dissolved in demineralized water, pH 6.4 with HCl

**Table 3:** Mean ± standard deviation values of Δ*E*_00_ according to dentin condition and adhesive protocol.

	Sound Dentin	Eroded Dentin	Mean (IDS)
No IDS	2.29 (0.51)	2.73 (0.37)	2.51 (0.49) A
IDS	4.47 (0.91)	4.79 (0.89)	4.63 (0.89) B
Mean (Dentin condition)	3.38 (1.33)	3.76 (1.25)	-

Two-way ANOVA: dentin condition (*p* = 0.100); IDS (*p* < 0.001); interaction (*p* = 0.785). Uppercase letters indicate comparisons between adhesive protocol levels: A = No IDS and B = IDS. Different uppercase letters indicate statistically significant differences (Tukey test, α = 0.05).

**Table 4:** Two-way ANOVA results for Δ*E*_00_.

Source of Variation	Sum of Squares	df	Mean Square	F	*p* Value	Partial η^2^	Effect Estimate (95% CI)
Dentin condition	1.433	1	1.433	2.855	0.100	0.073	0.38 (−0.07 to 0.83)
IDS	45.135	1	45.135	89.938	<0.001	0.714	2.12 (1.67 to 2.57)
Dentin condition × IDS	0.038	1	0.038	0.075	0.785	0.002	−0.12 (−1.03 to 0.79)
Residual	18.066	36	0.502	—	—	—	—
Total	64.672	39	—	—	—	—	—

**Table 5:** Baseline and final CIELAB coordinates according to dentin condition and adhesive protocol.

Coordinate	Time Point	S-NoIDS	S-IDS	E-NoIDS	E-IDS
L*	Baseline	87.17 ± 2.69	87.89 ± 2.36	81.23 ± 2.91	85.44 ± 4.98
	Final	84.54 ± 2.59	81.61 ± 2.83	77.58 ± 2.89	78.68 ± 4.16
a*	Baseline	0.21 ± 0.55	−0.12 ± 0.82	0.24 ± 0.72	−0.94 ± 0.52
	Final	−0.48 ± 0.65	0.16 ± 0.94	0.34 ± 0.51	−0.26 ± 0.36
b*	Baseline	15.95 ± 1.32	15.91 ± 1.23	17.01 ± 1.85	15.18 ± 1.24
	Final	14.86 ± 1.17	15.69 ± 2.04	16.28 ± 1.26	15.62 ± 0.93

**Table 6:** Mean ± standard deviation values of ΔL*, Δa*, and Δb* according to dentin condition and adhesive protocol.

Variable	S-NoIDS	S-IDS	E-NoIDS	E-IDS	Mean (Sound)	Mean (Eroded)	*p*	Mean (No IDS)	Mean (IDS)	*p*	Interaction (*p*)
ΔL*	−2.63 (1.44)	−6.28 (1.92)	−3.65 (0.41)	−6.76 (1.63)	−4.46	−5.21	0.114	−3.14 A	−6.52 B	<0.001	0.567
Δa*	−0.68 (0.53)	0.12 (0.94)	0.13 (0.54)	0.68 (0.66)	−0.28 A	0.41 B	0.003	−0.28 A	0.40 B	0.004	0.573
Δb*	−1.09 (0.76)	−0.22 (1.60)	−0.73 (1.27)	0.45 (1.21)	−0.66	0.12	0.201	−0.91 A	0.12 B	0.014	0.694

Different uppercase letters indicate statistically significant differences within the same factor (Tukey test, α = 0.05).

**Table 7:** Two-way ANOVA results for changes in CIELAB coordinates.

Outcome	Effect	F(1, 36)	*p*	Partial η^2^	Estimated Difference (95% CI)
ΔL*	Dentin condition	2.628	0.1138	0.068	−0.75 (−1.69 to 0.19)
	IDS	53.428	<0.0001	0.598	−3.38 (−4.32 to −2.44)
	Dentin condition × IDS	0.335	0.5660	0.009	0.54 (−1.34 to 2.41)
Δa*	Dentin condition	9.927	0.0033	0.216	0.69 (0.24 to 1.13)
	IDS	9.583	0.0038	0.210	0.68 (0.23 to 1.12)
	Dentin condition × IDS	0.329	0.5700	0.009	−0.25 (−1.13 to 0.63)
Δb*	Dentin condition	1.681	0.2030	0.045	0.51 (−0.29 to 1.31)
	IDS	6.712	0.0137	0.157	1.02 (0.22 to 1.82)
	Dentin condition × IDS	0.154	0.6970	0.004	0.31 (−1.29 to 1.91)

## Data Availability

The original contributions presented in this study are included in the article. Further inquiries can be directed to the corresponding author.

## Abbreviations

The following abbreviations are used in this manuscript:

IDS	Immediate dentin sealing
NoIDS	Without immediate dentin sealing
S-NoIDS	Sound dentin without immediate dentin sealing
S-IDS	Sound dentin with immediate dentin sealing
E-NoIDS	Eroded dentin without immediate dentin sealing
E-IDS	Eroded dentin with immediate dentin sealing
CAD/CAM	Computer-aided design/computer-aided manufacturing
CIELAB	Commission Internationale de l’Eclairage L*a*b* color space
Δ*E*_00_	CIEDE2000 color difference
ΔL*	Change in luminosity
Δa*	Change in the red–green chromatic coordinate
Δb*	Change in the yellow–blue chromatic coordinate
PT	Perceptibility threshold
AT	Acceptability threshold
ANOVA	Analysis of variance
PVC	Polyvinyl chloride
LED	Light-emitting diode
